# Natural Compounds Oridonin and Shikonin Exhibit Potentially Beneficial Regulatory Effects on Select Functions of Microglia

**DOI:** 10.3390/brainsci14040328

**Published:** 2024-03-28

**Authors:** Bridget K. Greuel, Dylan E. Da Silva, Victoria N. Robert-Gostlin, Andis Klegeris

**Affiliations:** Laboratory of Cellular and Molecular Pharmacology, Department of Biology, University of British Columbia Okanagan Campus, Kelowna, BC V1V 1V7, Canadavictoriarobertgostlin@gmail.com (V.N.R.-G.)

**Keywords:** Alzheimer’s disease, cytokines, glia, NLRP3 inflammasomes, neuroinflammation, neuroprotection, neurotoxicity, phagocytosis, reactive oxygen species

## Abstract

Accumulating evidence indicates that the adverse neuroimmune activation of microglia, brain immunocytes that support neurons, contributes to a range of neuroinflammatory disorders, including Alzheimer’s disease. Correcting the abnormal functions of microglia is a potential therapeutic strategy for these diseases. Nucleotide-binding domain leucine-rich repeat and pyrin domain-containing receptor (NLRP) 3 inflammasomes are implicated in adverse microglial activation and their inhibitors, such as the natural compounds oridonin and shikonin, reduce microglial immune responses. We hypothesized that some of the beneficial effects of oridonin and shikonin on microglia are independent of their suppression of NLRP3 inflammasomes. Murine and human microglia-like cells were stimulated with bacterial lipopolysaccharide (LPS) only, which did not induce NLRP3 inflammasome activation or the resulting secretion of interleukin (IL)-1β, allowing for the identification of other anti-inflammatory effects. Under these experimental conditions, both oridonin and shikonin reduced nitric oxide (NO) secretion and the cytotoxicity of BV-2 murine microglia towards HT-22 murine neuronal cells, but upregulated BV-2 cell phagocytic activity. Only oridonin inhibited the secretion of tumor necrosis factor (TNF) by stimulated BV-2 microglia, while only shikonin suppressed the respiratory burst response of human HL-60 microglia-like cells. This observed discrepancy indicates that these natural compounds may have different molecular targets in microglia. Overall, our results suggest that oridonin and shikonin should be further investigated as pharmacological agents capable of correcting dysfunctional microglia, supporting their potential use in neuroinflammatory disorders.

## 1. Introduction

Neuroinflammatory mechanisms contribute to numerous neurological conditions, including neurotrauma, stroke, and neurodegenerative disorders such as Alzheimer’s disease (AD). Microglia, the resident innate immune cells of the central nervous system (CNS), orchestrate the neuroimmune responses in most, if not all, of these conditions. Under such neuropathological conditions, microglia acquire diverse reactive phenotypes and are believed to actively contribute to the pathophysiological processes during at least the specific phases of neurodegenerative diseases (reviewed in [1,2]). For example, in AD, a gradual transition from a homeostatic to a disease-associated microglia (DAM) state has been documented, which is characterized by the downregulation of homeostatic genes, a compromised phagocytic function, and the upregulated secretion of pro-inflammatory and neurotoxic factors (reviewed in [1,3,4]). Several pharmacological approaches aimed at suppressing the reactivity and the harmful actions of microglia in AD have been elucidated, including the use of such broadly acting medicines as non-steroidal anti-inflammatory drugs as well as biologics targeting specific inflammatory mechanisms, including the tumor necrosis factor (TNF)-neutralizing etanercept; however, these approaches have not produced clinically significant beneficial results [5,6]. More recently, microglia-specific signaling pathways—for example, those involving p38 alpha mitogen-activated protein kinase (MAPK) or the nucleotide-binding oligomerization domain-like receptor family, pyrin domain-containing (NLRP) 3 inflammasome—have been suggested as targets for the development of AD therapeutics [7,8]. NLRP3 inflammasome components are highly expressed by microglia and the assembly of this macromolecular complex has been reported in this CNS cell type (reviewed in [8,9]). The activation of the NLRP3 inflammasome classically requires the sequential actions of a priming agent, such as the toll-like receptor (TLR) 4 agonist lipopolysaccharide (LPS), and a specific stimulus, such as adenosine triphosphate (ATP). The assembly of a functional NLRP3 inflammasome leads to the conversion of procaspase-1 into active caspase-1, resulting in the production of pro-inflammatory cytokines interleukin (IL)-1β and IL-18 from their respective precursors (reviewed in [10]). Notably, compared with control subjects, elevated levels of cleaved caspase-1 have been measured in hippocampal and cortical lysates from AD and mild cognitive impairment (MCI) patients [11], while increased concentrations of IL-1β have been observed in the cerebrospinal fluid (CSF) of AD patients compared with control subjects without significant differences in the plasma levels of this cytokine [12]. All these observations are consistent with chronic inflammasome activation in AD brains. Furthermore, the assembly of NLRP3 inflammasomes has been observed in both human and murine microglia and their inhibition induces protective and anti-inflammatory effects in this cell type, including not only reduced production of IL-1β and IL-18, but also increased IL-4 secretion, lowered NO release, and enhanced phagocytic activity [11,13]. Therefore, a diverse range of therapeutic strategies and agents targeting NLRP3 inflammasomes has been proposed for the treatment of AD, including several bioactive compounds derived from herbal medicines (reviewed in [9,14]).

Oridonin and shikonin are two plant-derived compounds that have been reported to exhibit NLRP3 inflammasome inhibitory activity in several different cell types. Their inflammasome-targeting mechanisms of action are different. Thus, oridonin binds covalently to NLRP3, preventing its interaction with NIMA-related kinase (NEK) 7, which is a required step in inflammasome assembly [14,15]. Meanwhile, shikonin directly inhibits caspase-1 enzymatic activity and the oligomerization of apoptosis-associated speck-like proteins containing caspase recruitment domains (ASC), which is necessary for NLRP3 inflammasome activation [16]. All these actions block the conversion of pro-IL-1β to its mature form, leading to a reduced production of this pro-inflammatory cytokine in various cell types, including reactive microglia and other mononuclear phagocytes [15,16,17]. In addition to their anti-inflammatory potential, NLRP3 inflammasome inhibitors exhibit cytotoxic properties that make them attractive anti-cancer drug candidates [14,18,19]. However, cytotoxicity is an undesirable feature for the development of anti-inflammatory agents intended for the long-term treatments of slowly progressing neuroinflammatory and neurodegenerative disorders. Therefore, identifying anti-inflammatory properties and targets that can be manipulated without inflicting cellular damage is a valid strategy for the development of anti-AD drugs.

Interestingly, both oridonin and shikonin have been reported to possess anti-inflammatory activity that is independent of their direct effects on NLRP3 inflammasomes. For example, oridonin has been shown to downregulate nuclear factor (NF)-κB protein levels in LPS-stimulated RAW 264.7 murine macrophages and block the translocation of this transcription factor from the cytosol to the nucleus [20,21]. Similarly, shikonin suppresses the LPS-stimulated DNA-binding activity of NF-κB in primary rat microglia, and inhibits the phosphatidylinositol 3-kinase (PI3K)/Akt signaling pathway and phosphorylation of extracellular signal-regulated kinase (ERK) 1/2 in this cell type [22]. Therefore, we hypothesized that at least some of the beneficial anti-inflammatory actions of oridonin and shikonin on microglia could be mediated by NLRP3-independent mechanisms. We tested this hypothesis by investigating the effects of both these natural compounds on microglia-like cells that were immune-activated by LPS alone. The exposure of these cells to LPS alone did not induce IL-1β production and, hence, the assembly of NLRP3 inflammasomes.

## 2. Materials and Methods

### 2.1. Reagents and Cells

Oridonin (7a,20-epoxy-1a,6b,7,14-tetrahydroxy-kaur-16-en-15-one) and shikonin ((R)-5,8-dihydroxy-2-(1-hydroxy-4-methylpent-3-en-1-yl) naphthalene-1,4-dione) were purchased from Cayman Chemicals (Ann Arbor, MI, USA). ELISA development kits for murine IL-1β and TNF were purchased from PeproTech (Embrun, ON, Canada). Fluorescein isothiocyanate (FITC) externally labeled 1 μm fluorescent polystyrene latex beads were purchased from Bangs Laboratories (Fishers, IN, USA). The following reagents were obtained from Sigma Aldrich (Oakville, ON, Canada): bisbenzimide (Hoechst 33258), dimethyl sulfoxide (DMSO), N-formylmethionine-leucyl-phenylalanine (fMLP), luminol sodium salt, LPS, and N,N-dimethylformamide (DMF). Calf bovine serum (CBS), Dulbecco’s modified Eagle medium nutrient mixture F12/Ham (DMEM-F12), phenol red-free DMEM-F12, 0.05% and 0.025% trypsin with ethylenediaminetetraacetic acid (EDTA), and all other reagents were obtained from Thermo Fisher Scientific (Ottawa, ON, Canada). BV-2 murine microglial cells were obtained from Dr G Garden, Department of Neurology, University of Washington (Seattle, WA, USA). HT-22 murine hippocampal neuronal cells were donated by Dr T Wenzel (University of Saskatchewan, Saskatoon, SK, Canada). HL-60 human monocytic cells were purchased from American Type Culture Collection (ATCC, Manassas, VA, USA). All cells used in this study were cultured in T75 cell culture flasks containing DMEM-F12 supplemented with 10% CBS and the antibiotics, which were penicillin (100 U/mL) and streptomycin (100 μg/mL), at 37 °C in humidified 5% CO_2_ and a 95% air atmosphere.

### 2.2. Secretion of Cytotoxins and Cytokines by BV-2 Murine Microglia 

A previously published procedure with modifications was used to assess the cytotoxic effects of BV-2 cells towards murine neuronal cells [23]. BV-2 microglia were detached from plastic by treating cell cultures with 0.25% trypsin–EDTA. Cells were seeded in 24-well plates at 2 × 10^5^ cells/mL in 0.5 mL DMEM-F12 containing 5% CBS. Cells were allowed to adhere for 24 h, then their supernatants were aspirated and replaced with a fresh medium at 37 °C. The cells were treated with oridonin (1–4 μM), shikonin (40–400 nM), or their vehicle solution, DMSO, for 30 min followed by 24 h incubation in either the presence or absence of LPS (100 ng/mL). The concentration of LPS used in this and other assays was selected based on preliminary experiments employing a range of LPS concentrations, which identified the optimal conditions for cell stimulation, including the maximal release of inflammatory mediators such as TNF and NO. Next, cell-free supernatants were collected and transferred to HT-22 neuronal cell cultures or used to measure concentrations of IL-1β and TNF using ELISAs according to the instructions provided by the manufacturer (PeproTech). BV-2 cell viability was measured using the MTT assay. To investigate the effect of oridonin and shikonin on the secretion of cytotoxins by BV-2 microglia, 400 μL of cell-free supernatants were transferred to separate wells containing NSC-34 neuronal cells that had been seeded 24 h earlier at 2 × 10^5^ cells/mL in 400 μL of DMEM-F12 containing the antibiotics and 5% CBS. After 72 h incubation, NSC-34 neuronal cell viability was measured using the MTT assay. 

### 2.3. NO Release by BV-2 Murine Microglia

Concentrations of nitrite, the breakdown product of NO, were measured by adding 50 μL of Griess reagent (1% *w*/*v* sulfanilamide, 2.5% *v*/*v* phosphoric acid, and 0.1% *w*/*v* N-(1-naphthyl)ethylenediamine dihydrochloride in water) to 50 μL of BV-2 cell supernatants and measuring optical densities at 570 nm using a FLUOstar Omega microplate reader from BMG Biotech (Ortenberg, Germany). Concentrations of nitrite in cell culture supernatants were interpolated by comparisons with nitrite standard solutions.

### 2.4. Phagocytic Activity of BV-2 Murine Microglia

The phagocytosis of latex beads by BV-2 cells was studied as previously described with minor modifications [24]. BV-2 microglia (5 × 10^4^ cells/mL) were seeded for 24 h in four-chambered glass-bottom dishes in 500 μL of DMEM-F12 containing 5% CBS and antibiotics and allowed to adhere for 24 h. After replacing the cell culture media with fresh, the cells were treated with oridonin (4 μM), shikonin (400 nM), or their vehicle solution (DMSO) for 30 min prior to stimulation with LPS (400 ng/mL) for 24 h. Control cultures were exposed to phosphate-buffered saline (PBS) instead. After replacing the cell culture media with fresh medium, FITC-labeled latex beads were added to each well at a 10:1 bead-to-cell ratio and the plates were incubated for 1 h. External beads were washed away using PBS and the cells were fixed using 500 μL of cold 70% ethanol for 5 min before being washed again with PBS. Immediately prior to imaging, a bisbenzimide (2 μg/mL) nuclear stain was added to all wells in 500 μL PBS. A Zeiss AxioObserver.Z1 inverted widefield fluorescence microscope and Zen 2.0 acquisition software were used to record fluorescence intensities at an excitation/emission of 365/445 nm for bisbenzimide and 474/537 nm for the fluorescent beads. A blind analysis of the images by an investigator not knowing the experimental conditions was then completed using NIH ImageJ software (version 1.53, imagej.net, accessed on 14 April 2021). 

### 2.5. Release of Reactive Oxygen Species by HL-60 Human Microglia-Like Cells

The respiratory burst response was induced in differentiated HL-60 human monocytic cells to study the effects of oridonin and shikonin on the secretion of reactive oxygen species (ROS) by microglia-like cells [25]. First, HL-60 cells (3 × 10^6^ cells) were plated in 10 cm^2^ culture dishes and incubated in 15 mL of the DMEM-F12 medium containing the antibiotics, 10% CBS, and 1.3% *v*/*v* DMSO for six days, which has previously been reported to upregulate their expression of NADPH oxidase components [26]. The cells were resuspended at 1 × 10^6^ cells/mL in a fresh DMEM-F12 medium containing the antibiotics and 10% CBS and 1 mL per well of cell suspension was added to 24-well plates. Cells were treated for 30 min with oridonin (1–4 µM), shikonin (40–400 nM), or their vehicle solution and primed with LPS (500 ng/mL) for 24 h. In total, 500 µL of each sample were used to conduct the MTT cell viability assay, while the remaining cells were transferred to a phenol red-free DMEM-F12 medium containing the antibiotics and 2% CBS. Next, 85 µL of each sample containing differentiated HL-60 cells at 1 × 10^6^ cells/mL were transferred to a 96-well plate. The chemiluminescent signal in each well was measured for 21 min after sequential injections of 10 µL of a luminol solution (0.85 mg/mL in PBS) and 5 µL fMLP (20 µM in PBS), which is an established inducer of the respiratory burst of phagocytes [27]. 

### 2.6. Cell Viability Assay

Cell viability was assessed using an assay that measured the reduction of MTT to insoluble colored formazan crystals by viable cells as previously described [24]. Cell cultures were incubated with MTT (0.5 mg/mL) at 37 °C for 1 h. Formazan crystals were dissolved by adding a volume of a sodium lauryl sulfate (20% *w*/*v*)/DMF (50% *v*/*v*) solution equal to that of the culture medium present in the well and then shaking the plates for 3 h. Optical densities at 570 nm were measured and the cell viability data were presented as a percentage compared with the values obtained from cells incubated in the growth medium alone.

### 2.7. Statistical Analysis

The statistical analysis was completed using GraphPad PRISM software (version 10.0, GraphPad Software Inc., La Jolla, CA, USA). A randomized block one-way analysis of variance (ANOVA) was performed to determine the significance of the differences in the obtained data. Where significant, the ANOVA was followed by Dunnett’s or Tukey’s post hoc tests. Data were presented as the mean ± standard error of the mean (SEM). The statistical significance was established as *p* < 0.05. 

## 3. Results

According to a two-signal model of activation of the NLRP3 inflammasome, it becomes fully functional in the presence of both a priming agent and an activating signal [28]. As we were interested in the NLRP3-independent microglia modulatory effects of oridonin and shikonin, we used LPS as the sole stimulus for microglial cells. We used BV-2 murine microglia as the model cells in most of our assays because they have been shown to respond to stimulation with LPS alone by secreting inflammatory cytokines, NO, and a mixture of cytotoxins capable of killing neuronal cells [23]. DMSO-differentiated HL-60 human cells were selected to model microglial ROS secretion because the respiratory burst response of these cells can be primed by incubation with LPS alone [25].

**Figure 1:**
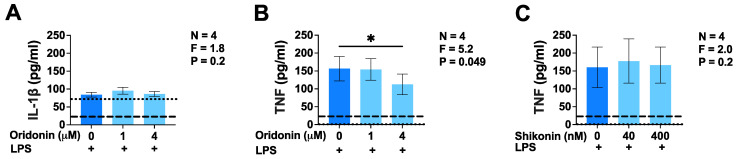
Effects of oridonin and shikonin on the secretion of IL-1β and TNF by LPS-stimulated BV-2 murine microglia. Two different concentrations of either drug or the vehicle solution (DMSO) were added to BV-2 cells 30 min before their stimulation with LPS (100 ng/mL). Following 24 h incubation, concentrations of IL-1β (**A**) or TNF (**B**,**C**) in cell-free supernatants were determined using ELISAs. Data from four independent experiments performed on separate days are presented as mean ± SEM. * *p* < 0.05 according to Dunnett’s post hoc test. F- and *p*-values displayed on the graphs were calculated using a one-way randomized block ANOVA. The dashed lines indicate the detection limits of the ELISAs. The dotted lines represent the baseline concentrations of IL-1β (A) and TNF (**B**,**C**) measured in the supernatants of unstimulated BV-2 murine microglia incubated in the absence of LPS.

**Figure 2:**
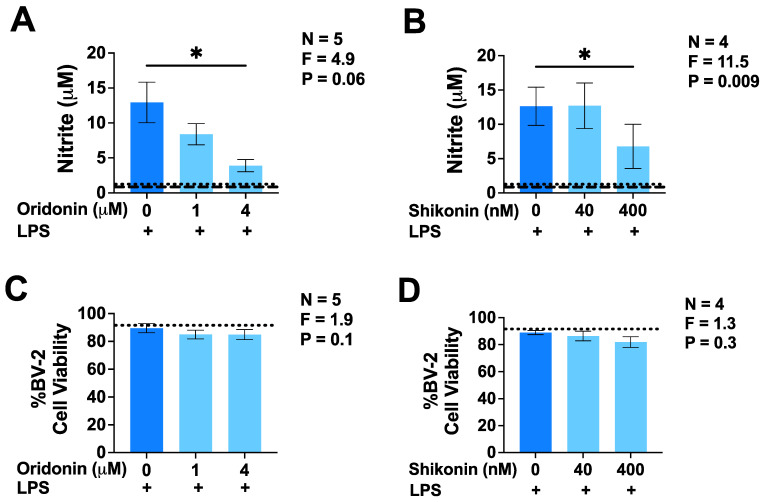
Effects of oridonin and shikonin on the NO secretion and viability of LPS-stimulated BV-2 murine microglia. Two different concentrations of either drug or the vehicle solution were added to BV-2 cells 30 min before their stimulation with LPS (100 ng/mL). Following 24 h incubation, concentrations of nitrite in cell-free supernatants were measured using the Griess assay (**A**,**B**) and the viability of BV-2 cells was measured using the MTT assay (**C**,**D**). Data from four to five independent experiments performed on separate days are presented as mean ± SEM. * *p* < 0.05 according to Dunnett’s post hoc test. F- and *p*-values displayed on the graphs were calculated using a one-way randomized block ANOVA. The dashed lines indicate the detection limit of the Griess assay. The dotted lines represent the baseline nitrite concentrations (**A**,**B**) or cell viability values (**C**,**D**) obtained from unstimulated BV-2 murine microglia incubated in the absence of LPS.

Preliminary studies with LPS-stimulated BV-2 cell cultures were performed to determine the maximal non-toxic concentration of the two drugs. These were identified as 4 µM for oridonin and 400 nM for shikonin. Figure 1A illustrates that unstimulated BV-2 murine microglia secreted low levels of IL-1β that were not upregulated by exposure to LPS for 24 h, confirming the inability of this TLR4 agonist alone to activate NLRP3 inflammasomes. Unstimulated BV-2 microglia did not secrete detectable levels of TNF; however, the secretion of this cytokine was induced by LPS alone (Figure 1B,C). Adding oridonin to BV-2 cells before their activation by LPS caused a concentration-dependent inhibition of TNF secretion, an effect that was not observed when shikonin was used to pretreat BV-2 cells (Figure 1B,C). However, oridonin did not inhibit or upregulate the secretion of IL-1β by LPS-stimulated BV-2 cells (Figure 1A). The lack of the effect of shikonin on TNF secretion could not be attributed to the 10-fold lower concentrations used for this drug because, at this range, its inhibitory effect on NO production by LPS-stimulated BV-2 microglia was significant and similar to that of oridonin (Figure 2A,B). Notably, unstimulated BV-2 cells, similar to TNF, did not produce detectable NO. Additionally, we confirmed the lack of toxic effects of oridonin and shikonin at the selected concentrations on LPS-stimulated BV-2 microglia (Figure 2C,D); therefore, the inhibitory effects of neither of these two drugs on microglial functions were due to decreased cell viability.

**Figure 3:**
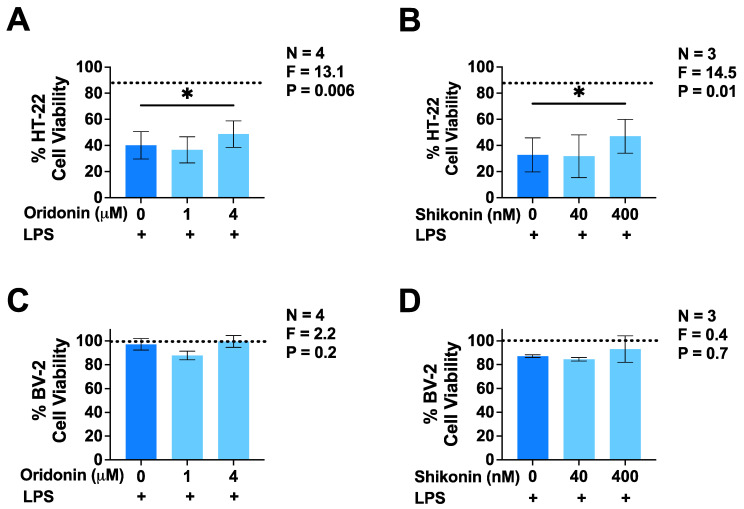
Effects of oridonin and shikonin on the cytotoxicity of LPS-stimulated BV-2 murine microglia cells towards HT-22 murine neuronal cells. Two different concentrations of either drug or the vehicle solution were added to BV-2 cells 30 min before their stimulation with LPS (100 ng/mL). Following 24 h incubation, BV-2 microglia supernatants were transferred onto HT-22 neuronal cell cultures and the viability of BV-2 microglial cells was measured using the MTT assay (**C**,**D**). Following an additional 72 h incubation period with BV-2 cell supernatants, the viability of HT-22 neuronal cells was measured using the MTT assay (**A**,**B**). Data (mean ± SEM) from three to four independent experiments performed on separate days are presented as a percent of the values obtained from HT-22 neuronal cells exposed to a fresh culture medium only (**A**,**B**). * *p* < 0.05 according to Dunnett’s post hoc test. F- and *p*-values displayed on the graphs were calculated using a one-way randomized block ANOVA. Dotted lines represent the mean baseline viability of HT-22 neuronal cells incubated in supernatants from unstimulated BV-2 microglial cells (**A**,**B**) or viability of unstimulated BV-2 cells (**C**,**D**) incubated in the absence of LPS.

Considering that our data showed an inhibitory activity of both oridonin and shikonin on the production of TNF and NO, which could potentially be neurotoxic [4], we studied the protective effect of these compounds on the cytotoxicity of LPS-stimulated BV-2 microglia towards HT-22 murine neuronal cells. To examine the potential toxicity of BV-2 microglia secretions, we transferred supernatants from unstimulated BV-2 microglia to HT-22 cell cultures and observed only a negligible effect on the viability of neuronal cells; however, supernatants from LPS-stimulated BV-2 cells reduced HT-22 neuronal cell viability to less than 40% when compared with cells incubated in a fresh cell culture medium (Figure 3A,B). Both oridonin (4 µM) and shikonin (400 nM), when added to BV-2 cells before stimulation with LPS, significantly reduced the cytotoxic action of microglial cell supernatants. This protective effect was not due to the reduced viability of BV-2 cells in the presence of either of the two drugs at the concentrations tested (Figure 3C,D).

**Figure 4:**
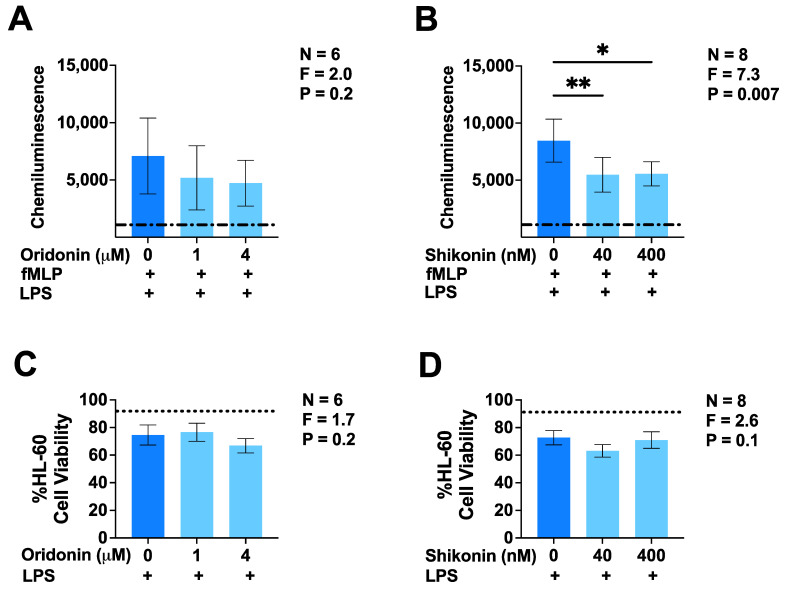
Effects of oridonin and shikonin on the secretion of ROS by differentiated HL-60 human microglia-like cells. Two different concentrations of either drug or the vehicle solution were added to DMSO-differentiated HL-60 cells 30 min before their priming with LPS (500 ng/mL). Following 24 h incubation, cells were stimulated with fMLP (1.2 µM) and luminol-dependent chemiluminescence was measured over a 25 min period (**A**,**B**). The viability of differentiated and LPS-primed HL-60 microglia-like cells plated in separate wells was measured using the MTT assay (**C**,**D**). Data from six or eight independent experiments performed on separate days are presented as mean ± SEM. * *p* < 0.05 and ** *p* < 0.01 according to Dunnett’s post hoc test. F- and *p*-values displayed on the graphs were calculated using a one-way randomized block ANOVA. The dash-dotted lines represent the luminescence signal from unprimed and fMLP-stimulated HL-60 microglia-like cells (**A**,**B**). The dotted lines represent the mean baseline viability of unprimed HL-60 cells incubated in the absence of LPS (**C**,**D**).

In the next series of experiments, we used a different microglia model—differentiated HL-60 human monocytic cells, which are known to express high levels of NADPH-dependent oxidase [26]—to assess whether oridonin and shikonin had an inhibitory effect on ROS production. The exposure of HL-60 cells to LPS for 24 h enhanced their ROS production in response to stimulation by fMLP (Figure 4A,B). The addition of oridonin before LPS priming led to only a trend towards reduced ROS production by HL-60 cells. Meanwhile, shikonin at both test concentrations significantly inhibited fMLP-induced ROS production by this cell type. Furthermore, we confirmed that neither of the drugs had a significant effect on the viability of HL-60 cells under the experimental conditions of this study (Figure 4C,D); therefore, the observed inhibitory effects were not caused by decreased cell viability.

**Figure 5:**
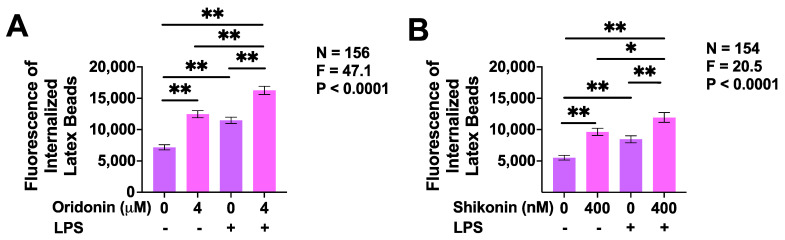
Effects of oridonin and shikonin on the phagocytosis of latex beads by unstimulated and LPS-stimulated BV-2 murine microglia. Oridonin (**A**) or shikonin (**B**), or the vehicle solution was added to BV-2 cells for 30 min, followed by 24 h incubation in the presence or absence of LPS (400 ng/mL). Fluorescent latex beads were added at a 10:1 bead-to-cell ratio for 1 h and fluorescence intensity of beads in live cells was measured using a widefield microscope. Data from four independent experiments (N = 154 or 156 cells total for each experimental condition) are presented as mean ± SEM. * *p* < 0.05 and ** *p* < 0.01 according to Tukey’s post hoc test. F- and *p*-values displayed on the graphs were calculated using a one-way ANOVA.

Clearance of cell debris, abnormal proteins, and pathogens by phagocytosis is considered to be one of the protective functions of microglia. Therefore, we studied the effect of oridonin and shikonin on the phagocytic activity of unstimulated and LPS-stimulated BV-2 microglia. LPS upregulated the phagocytosis of latex beads by BV-2 cells that had not been exposed to either of the drugs as well as by the cells that had been treated with oridonin (4 µM) or shikonin (400 nM) (Figure 5). Similarly, both oridonin (Figure 5A) and shikonin (Figure 5B) upregulated the phagocytosis of latex beads by unstimulated BV-2 cells and significantly enhanced the already elevated phagocytic activity of LPS-stimulated BV-2 microglial cells.

## 4. Discussion

To investigate the NLRP3 inflammasome-independent effects of oridonin and shikonin on immune-activated microglia, we selected a cell culture system that has been shown to not involve the assembly and activation of this inflammasome. We first confirmed, using BV-2 murine microglia, previous observations that LPS alone did not activate NLRP3 inflammasomes in murine microglia-like cells. Sha et al. [29] demonstrated that murine bone-marrow-derived macrophages (BMDMs) secreted a basal level of IL-1β and that this secretion was not upregulated by LPS alone. These researchers further confirmed that the NLRP3 inflammasome was not assembled in LPS-activated BMDMs and that its activation required a secondary stimulus, in their case, bacterial RNA. Similar observations were made for murine primary microglia in addition to BMDMs, where NLRP3 inflammasomes were not activated, and the basal levels of IL-1β secretion were not upregulated by LPS alone, but became functional after the exposure of cells to several secondary stimuli, including ATP and the antibiotic nigericin [30]. As we were interested in the protective effects of oridonin and shikonin at non-cytotoxic concentrations, we performed preliminary experiments that identified a notable difference between these two compounds in terms of their maximum non-toxic concentrations. This observation was in agreement with the cytotoxicity data collected by others for oridonin and shikonin using cancer cell lines [18,19]. For example, oridonin reduced the viability of human small cell lung cancer cells (H1688) at concentrations above 10 µM, which aligned with the 1–4 µM range selected for this drug in our study. Notably, oridonin at the non-toxic 5 µM concentration inhibited the migration of H1688 cells without affecting their viability [18]. Much lower cytotoxic concentrations have been reported for shikonin, which inhibited the proliferation of 14 out of 15 different cancer cell lines tested with IC50 values below 10 µM. These data correspond well with our observed cytotoxicity of shikonin towards microglial cells at above 0.4 µM. 

Table 1 summarizes effects of oridonin and shikonin applied at non-toxic concentrations on the microglia-like cell functions assessed in this study. It should be noted that previous research used both oridonin and shikonin to treat and inhibit the pro-inflammatory functions of both microglia and macrophages at much higher concentrations, sometimes without reporting their effects on cell viability, which makes the interpretation of such data challenging (e.g., [31,32]). Nevertheless, earlier research supports some of the key observations of our study. Xu et al. [17] used primary rat microglia to demonstrate the inhibition of TNF secretion and inducible NO synthase (iNOS) expression by oridonin at concentrations similar to those used in this study (2 and 4 µM) without confirming its effect on cell viability. Nam et al. [22] found that the shikonin derivatives isobutyrylshikonin and isovalerylshikonin, both at 4 µM, reduced the secretion of NO and TNF by LPS-stimulated primary rat microglia and had no effect on the viability of these cells. Prasad et al. [33] reported an inhibitory effect of shikonin at non-toxic concentrations of 400 and 800 nM on NO and TNF production by LPS-stimulated BV-2 microglia. The inhibition of the LPS-induced secretion of both these mediators by shikonin at concentrations ranging from 0.5 to 2 µM has also been reported for RAW 264.7 murine macrophages [32]. Even though the viability of the cells exposed to such high concentrations of shikonin was not reported, this study identified NF-κB and Janus kinase (JAK) as the potential molecular targets for this compound. Our data aligned well with these previous observations, except for the lack of inhibition of TNF secretion by shikonin in our experiments. This discrepancy may have been due to the lower concentration range of shikonin we used because Prasad et al. [33] recorded less than 20% inhibition of TNF secretion at 400 nM, which was the concentration used in our study. This effect increased to approximately 40% at 800 nM, a concentration that was toxic to BV-2 cells in our study. 

Reactive microglia can secrete a broad range of potentially cytotoxic molecules, including NO and TNF, that can contribute to neuronal death observed in neurodegenerative diseases (reviewed in [4,34]). We have previously used LPS-stimulated BV-2 murine microglia to demonstrate their direct toxic effects on NSC-34 murine motor neuron-like cells [23]. In this study, we employed an HT-22 murine hippocampal neuronal cell line that better models the CNS neurons affected in AD to confirm the cytotoxic action of supernatants from BV-2 cells that had been stimulated with LPS alone. Adding either oridonin or shikonin before the exposure of BV-2 cells to the LPS stimulus significantly inhibited their cytotoxic action without affecting microglial cell viability. HT-22 cell death induced by LPS-stimulated BV-2 cells has already been reported by Ogunrinade et al. [35], who additionally demonstrated the activation of NF-κB and p38 MAPK in these cells. Liu et al. [36] also reported BV-2 cell supernatant toxicity towards HT-22 cells, which was induced by LPS priming followed by stimulation with amyloid β (Aβ1–42) and the resulting activation of NLRP3 inflammasomes. The same study used a specific inhibitor, CLI-095, to demonstrate the involvement of TLR4 in this BV-2 cell response. Therefore, TLR4, MAPK, and NF-κB activation are required for the LPS-induced cytotoxic response of BV-2 microglia. To the best of our knowledge, the effects of either oridonin or shikonin on microglia neurotoxic responses have not been studied before.

It is well established that the NADPH oxidase (NOX)-dependent production of large quantities of ROS by microglia is detrimental and could contribute to neurodegenerative-disease pathologies. This so-called respiratory burst response of microglia can be triggered by diverse stimuli, including Aβ and fMLP, and it is primed by TLR4 ligands, such as LPS, which upregulate the expression of NOX subunits [37,38,39]. While oridonin showed only a trend towards inhibition, pretreatment with shikonin significantly reduced ROS production by LPS-primed and fMLP-stimulated microglia-like cells. These observations aligned with the reported protective effects of oridonin on resident liver macrophages, Kupffer cells, which include the inhibition of LPS plus interferon-γ-induced ROS production [40]. This study also identified the inhibition of NF-κB as the molecular mechanism for this beneficial effect of oridonin. Even though the viability of cells was not monitored, the 5 µM effective concentration of oridonin appeared to not cause cytotoxicity because the secretion of two different cytokines was not affected.

Phagocytic activity is one of the protective functions of microglia and is essential for the removal of debris and pathogens as well as the regulation of brain development and synaptogenesis. Defective phagocytosis in AD brains could be one of the leading causes for the abnormal accumulation of Aβ aggregates and other waste products. The protective role of the microglial phagocytic function in AD is highlighted by the relatively recently identified association of increased late-onset AD risk with a mutation in triggering receptor expressed on myeloid cells (TREM) 2, which is a microglia-specific receptor that facilitates phagocytosis (reviewed in [41]). Therefore, the upregulation of the microglial phagocytic function by oridonin and shikonin observed in this study further supports the potential beneficial effects of these compounds in the context of AD pathophysiology. The regulation of microglial phagocytic activity by oridonin has not been studied before, but oridonin has been shown to upregulate this function in other types of phagocytes. For example, oridonin augmented the phagocytosis of apoptotic cells by U937 human monocytic cells [42] and RAW 264.7 murine macrophages [43]. The latter study also identified the direct activation of TLR4 as the molecular mechanism for the observed potentiation of phagocytic activity by oridonin. Furthermore, an in vitro cerebral hemorrhage model was used to demonstrate that shikonin upregulated the phagocytosis of red blood cells by primary murine microglia [31]; however, this compound was used at a very high concentration of 5 µM without reporting its effect on cell viability.

It has to be acknowledged that our study had significant methodological limitations, including the in vitro use of immortalized microglia-like cells, which have only a limited resemblance in terms of their functional responses to primary microglia residing in brain tissues. Furthermore, as only five cellular functions of microglia were studied, representing only a small subset of their full repertoire, our data were biased towards these particular functional responses of microglia and the associated molecular mechanisms. In addition, we only used a single microglial stimulus LPS, which is an established TLR4 agonist, to model select aspects of immune responses in this particular cell type that could be triggered under pathological conditions. Even though TLR4 plays a crucial role in numerous neuroimmune disorders [44], the use of disease-specific ligands of this receptor such as amyloid β as well as stimulants interacting with other receptors and signaling pathways relevant to various neuropathologies such as interferon-γ could provide additional insights into the molecular mechanisms engaged by oridonin and shikonin. Future studies assessing the effects of these drugs on microglia immune functions could also measure additional parameters of microglia activation, including the release of other pro- and anti-inflammatory cytokines, eicosanoids, proteases, lipases, and gliotransmitters that are used for communication between microglia and other CNS cell types. 

Despite these limitations, by systematically studying the effects of oridonin and shikonin on five distinct functions of microglia, we identified an overall pattern of their beneficial actions (Table 1). Importantly, these effects were not dependent on NLRP3 inflammasome activation in microglial cells even though both these compounds are often used as inflammasome inhibitors. The differential effects of oridonin and shikonin in two out of the five cellular assays employed indicate that these compounds might have overlapping but also distinct mechanisms of action on microglia. Previous research has identified that oridonin specifically can directly modulate TLR4 activity [43]. Furthermore, oridonin and shikonin have been implicated as direct or indirect modulators of JAK and NF-κB, the latter being linked to TLR4 signaling [32,40]. In addition, the observed inhibitory effects of oridonin and shikonin on the overall cytotoxicity of BV-2 microglia identify p38 and other MAPKs as the possible molecular targets of these drugs because these kinases have been shown to mediate the cytotoxic response of microglia [35,44,45]. The exact contribution of TLR4 and each of the other identified signaling molecules as potential mediators of the beneficial effects of oridonin and shikonin in microglia should be explored and confirmed by future mechanistic studies.

## 5. Conclusions

While the effects of oridonin and shikonin on some of the microglia-mediated neuroimmune mechanisms have been reported before, to the best of our knowledge, this study is the first that has been designed to assess the possible NLRP3-independent beneficial effects of these drugs on microglia. We applied drugs at non-toxic concentrations to microglial cells that were subsequently exposed to an immune stimulant not able to induce NLRP3 activation in these cells. We determined that under these experimental conditions, both oridonin and shikonin reduced NO secretion and overall cytotoxicity, while the phagocytic activity of microglial cells was enhanced. Only oridonin inhibited microglial TNF secretion, but shikonin suppressed ROS production. An analysis of this inhibitory pattern identified TLR4, JAK, MAPKs, and NF-κB as the potential molecular non-NLRP3 targets of the drugs studied in microglia, but this hypothesis requires future experimental proof. 

Our study demonstrates that oridonin and shikonin exhibit beneficial effects on immune-stimulated microglia by inhibiting the secretion of several potential neurotoxins (NO, ROS, and TNF) by reducing the cytotoxicity of their supernatants and by enhancing their phagocytic potential. All these actions are desirable outcomes in, for example, AD and other neurodegenerative disorders that are characterized by adverse microglia activation resulting in their neurotoxic activity and defective phagocytosis. Importantly, these favorable results are achieved by applying both drugs at concentrations that do not have a significant effect on the viability of microglial cells. As already mentioned above, the identification of non-cytotoxic candidates is an especially important aspect of drug development for slowly progressing neurodegenerative diseases that may require long-term pharmacological treatments. Further exploration of the NLRP3-independent mechanisms of action of oridonin and shikonin on microglia may identify new targets, allowing the selective manipulation of their dysregulated functions while preserving microglial homeostatic actions. It is important to note that human microglia and tissues should be used for the preclinical development of any drug candidates targeting microglia due to the emerging significant interspecies differences in neuroimmune mechanisms and immune responses of microglia in neurodegenerative diseases [1]. 

Overall, our research confirms both oridonin and shikonin can be used at non-toxic concentrations to beneficially regulate select immune functions of microglia in an NLRP3 inflammasome-independent manner. These compounds should be considered for future development as anti-neuroinflammatory drugs with potentially beneficial actions in AD.

## Figures and Tables

**Table 1:** Effects of oridonin and shikonin at non-toxic concentrations on select microglia-like cell functions.

Microglia Function	Oridonin 1–4 µM	Shikonin 40–400 nM
TNF secretion	↓	-
NO secretion	↓	↓
Cytotoxicity towards neuronal cells	↓	↓
ROS secretion	-	↓
Phagocytic activity	↑	↑

## Data Availability

The data presented in this study are available in the Supplementary Materials.

## Supplementary Materials

The following supporting information can be downloaded at: https://www.mdpi.com/article/10.3390/brainsci14040328/s1, Table S1: raw data.
